# Epitome of Diseases of the Skin

**Published:** 1885-11

**Authors:** 


EPITOME OF DISEASES OF THE SKIN, being an Abstract of a Course of Lectures delivered in the University of Pennsylvania during the session of 1883 and 1884. By Louis A. Duhring, M. D., Professor of Skin Diseases. Reported by Henry Wile, M. D., Clinical Assistant in the Department of Skin Diseases in the University Hospital. Philadelphia: J. B. Lippincott Company. 1886. Price 60 cents.

These lectures were originally reported for the *Medical News* by Dr. Henry Wile, now Lecturer on Skin Diseases in the Atlanta Medical College, and have since been gathered together and revised by the author, who says in his preface they were prepared with the view of presenting the subject in a simple, concise and practical a form as possible. In our judgment, he has accomplished his work right well and has given the profession a most valuable book.

